# Combined therapy of hypertensive nephropathy with ginkgo leaf extract and dipyridamole injection and antihypertensive drugs: A systematic review and meta-analysis

**DOI:** 10.1097/MD.0000000000025852

**Published:** 2021-05-14

**Authors:** Dinala Jialiken, Lichao Qian, Shuai Ren, Lihua Wu, Junyao Xu, Chong Zou

**Affiliations:** Affiliated Hospital of Nanjing University of Chinese Medicine, Nanjing, Jiangsu, China.

**Keywords:** ginkgo leaf extract and dipyridamole injection, hypertensive nephropathy, meta-analysis, systematic review

## Abstract

**Background:**

In recent years, the incidence rate of hypertensive nephropathy has been increasing quickly, which has been a major threat to people's health. Renin-angiotensin-aldosterone system blockers have certain curative effects. However, there are some patients having serious adverse reactions, and the benefit population is limited, so the treatment of hypertensive renal damage is necessary to have beneficial supplement. More and more clinical studies have shown that ginkgo leaf extract and dipyridamole injection (GDI) combined with antihypertensive drugs has achieved good results in the treatment of hypertensive renal damage. It is supposed to be a supplementary treatment in hypertensive nephropathy.

**Objectives:**

To systematically assess the efficacy and safety of GDI combined with antihypertensive drugs on hypertensive renal injury.

**Methods:**

Seven databases including PubMed, Cochrane Library, Embase, Wanfang database, China biomedical literature service system (Sino Med), VIP Chinese Sci-tech journal database (VIP), and China national knowledge internet (CNKI) were retrieved to collect randomized controlled trials (RCTs) in the experimental group containing combined therapy of hypertensive nephropathy with GDI and antihypertensive drugs. The retrieval time was from the establishment of database to July 8, 2020. Two researchers independently selected literature, extracted data, and evaluated the risk of bias in the study. The methodological quality was evaluated with Cochrane handbook and meta-analysis was performed with Stata 14.0 software.

**Results:**

Eight studies were included in this study which involved 556 patients. The meta-analyses indicated that, compared with using antihypertensive drugs alone, combined treatment of GDI with antihypertensive drugs can decrease 24-hour urinary total protein (weighted mean difference [WMD] –0.61, 95% confidence interval [CI]: –0.82, –0.39; *k* = 6, *P* ≤ .001), blood urea nitrogen (WMD –1.27, 95% CI: –2.45, –0.10; *k* = 6, *P* = .033, serum creatinine (WMD –29.50, 95% CI: –56.44, –2.56; number of estimates [*k*] = 6, *P* = .032).

**Conclusions:**

Our meta-analyses showed that GDI combined with antihypertensive drugs can improve the renal function of hypertensive patients with renal injury.

## 1 Introduction

Hypertension is one of the important causes of chronic kidney disease. In 2009, statistics from the American Nephrology Association showed that about 28% of patients with end-stage kidney disease (ESRD) were caused by hypertension. And the medical resources consumption reached about 5.6 billion US dollars.^[1]^ The Chinese kidney data system showed that ESRD patients caused by hypertension ranked the third, behind primary glomerular disease and diabetic nephropathy.^[2]^ Hypertensive nephropathy has been a serious threat to people's health which is caused by many factors, including poor control of blood pressure, inflammatory factors, etc.^[3–5]^ The pathogenesis of renal damage in hypertension mainly includes the increase of sympathetic nervous system activity, activation of renin-angiotensin system, the increase of salt load, genetic factors, the increase of resistance of anterior glomerular arterioles, and the increase of intraglomerular hypertension.^[6]^ Production of angiotensin (Ang) II is increased as the sympathetic nerve and renin-system are activated in patients with hypertensive nephropathy. Ang II is closely related to the dysfunction of plasma endothelium. It can stimulate the release of endothelin derived from vascular endothelial cells, cause the rise of vascular tension, promote the production of proteinuria, and aggravate the process of renal injury. According to the latest guidelines, Renin-angiotensin-aldosterone system blockers are recommended as drugs for the treatment of hypertensive renal damage, both of which have certain curative effects. However, there are some patients having serious adverse reactions, and the benefit population is limited, so the treatment of hypertensive renal damage is necessary to have beneficial supplement. At present, the treatment of hypertensive renal damage should not only emphasize the renal protection brought by pressure reduction, but we also have to pay attention to the specific renal protection of drugs.

Traditional Chinese medicine has its unique curative effect in the treatment of hypertensive renal damage.^[7–10]^ Ginkgo leaf extract and dipyridamole injection (GDI) is a common compound preparation.^[11]^ It is composed of total flavonoids of *Ginkgo biloba* L. and dipyridamole. It belongs to the fourth generation of *G biloba* L. preparations for intravenous application. *G biloba* L. is a plant with high medicinal value and has been used in hypertensive renal damage for a long time. *G biloba* L. extract has been proven to be useful to protect vascular endothelial cells, inhibit the activity of thromboxane A2, promote the production of prostacyclin *I*^2^ and nitric oxide, effectively inhibit the secretion of endothelin, thereby regulating vascular tension and improving tissue blood supply. In that way, it can play the role of reducing urinary protein and protecting renal function in patients with hypertensive renal damage so that it can delay the progress of hypertensive nephropathy.^[12–14]^ In recent years, more and more clinical studies have shown that GDI combined with antihypertensive drugs has achieved good results in the treatment of hypertensive renal damage.^[15–18]^ There may be differences in the related clinical research results. We conducted this meta-analysis by collecting all relevant available studies to review the efficacy and safety of GDI combined with antihypertensive drugs on hypertensive renal damage.

## 2 Materials and methods

### 2.1 Protocol registration

The protocol for this systematic review was registered (CRD42020141825), and our systematic review and meta-analysis was undertaken according to the Preferred Reporting Items for Systematic Reviews and Meta-Analyses (PRISMA) guidelines.^[19]^ All analyses were based on previous published studies, thus no ethical approval and patient consent were required.

### 2.2 Search strategy and study eligibility

We searched the following databases for relevant studies from the establishment of the database to July 8, 2020: PubMed, Cochrane Library, Embase, Wanfang database, China biomedical literature service system (Sino Med), VIP Chinese Sci-tech journal database (VIP), and China national knowledge internet (CNKI). And we collected randomized controlled trials (RCTs) of GDI combined with conventional therapy for hypertensive renal damage. The combination of subject words and free words was adopted in retrieval. Chinese key words included: “Yinxingdamo Zhusheye,” “Yinxingdamo,” “Zhusheyong Yinxingdamo,” “Xingding Zhusheye,” “Zhusheye Xingding,” “Gaoxueya Shensunhai,” “Gaoxueya,” “Gaoxueya Shenbing.” And English key words included: “Ginkgo leaf extract and Dipyridamole Injection,” “Ginkgo biloba Leaf,” “Ginkgo Extract,” “Ginkgo biloba L.,” “Yinxingdamo,” “Ginkgo-damole,” “GLEDI,” “GDI,” “Xingding,” “Hypertensive Nephropathy,” “Hypertensive Kidney Injury,” “Hypertension,” “Hypertensive Renal Damage,” and so on. At the same time, we also screened the references of the included articles to ensure that we can retrieve the relevant articles more comprehensively. (Tables S1 and S2 for the search strategy, Supplemental Digital Content).

### 2.3 Inclusion/exclusion criteria

Included articles should meet the following conditions: RCTs on hypertensive patients with renal damage; antihypertensive drugs as intervention measures in the control group, GDI plus antihypertensive drugs as intervention measures in the treatment group; primary outcome measures: serum creatinine (Scr), 24 hours urinary total protein (24 h UTP), blood urea nitrogen (BUN); secondary outcome measures: creatinine clearance rate (Ccr), mean arterial pressure (MAP), clinical efficacy. Included articles must include all or part of the above indicators from which we can extracted the relevant data. When multiple publications reported data from the same study, we included the study which had the largest sample size. We removed single case reports, animal studies, and basic science reports.

### 2.4 Data extraction and bias/quality assessment

Two investigators reviewed the literature independently, extracted data and then checked them crossly. When they met disagreements, the decision was made by discussion or judged by the third investigator. We extracted data including author, year of publication, patients’ characteristics, interventions, period of intervention, outcomes (24 h UTP, BUN, Scr, Ccr, MAP, clinical efficacy), adverse reactions, etc. When the data were incomplete or there was any uncertainty, we contacted the original author by email. We used the “risk of bias” evaluation tool in Cochrane Handbook to conduct the evaluation of quality, including the selection bias, implementation bias, measurement bias, follow-up bias, reporting bias, and bias from other sources. The following categories were used by us: high risk, low risk, and unclear.

### 2.5 Analysis

Relative risk (RR) and 95% confidence interval (CI) were used to represent the curative effect analysis statistics for a binary index. Weighted mean difference (WMD) and CI were used as the analysis statistic to represent continuous changes. We extracted RRs, WMDs, and 95% CIs from the publications, where available. When necessary, we calculated WMDs/CIs from original study data provided in the publication. We used *I*^2^ statistics and *P* value to evaluate the level of heterogeneity between the included studies. When *I*^2^ > 50% or *P* < .05, we chose the random effect model. Otherwise, the fixed effect model was chosen. We used sensitivity analysis to detect potential outliers by removing each estimate one at a time and recalculating the pooled estimates. Funnel plot and Egger test were used to determine whether there was publication bias. Meta-analysis was undertaken with Stata 14.0 software (StataCorp, TX).^[20]^

## 3 Results

### 3.1 Search results

**Figure 1:**
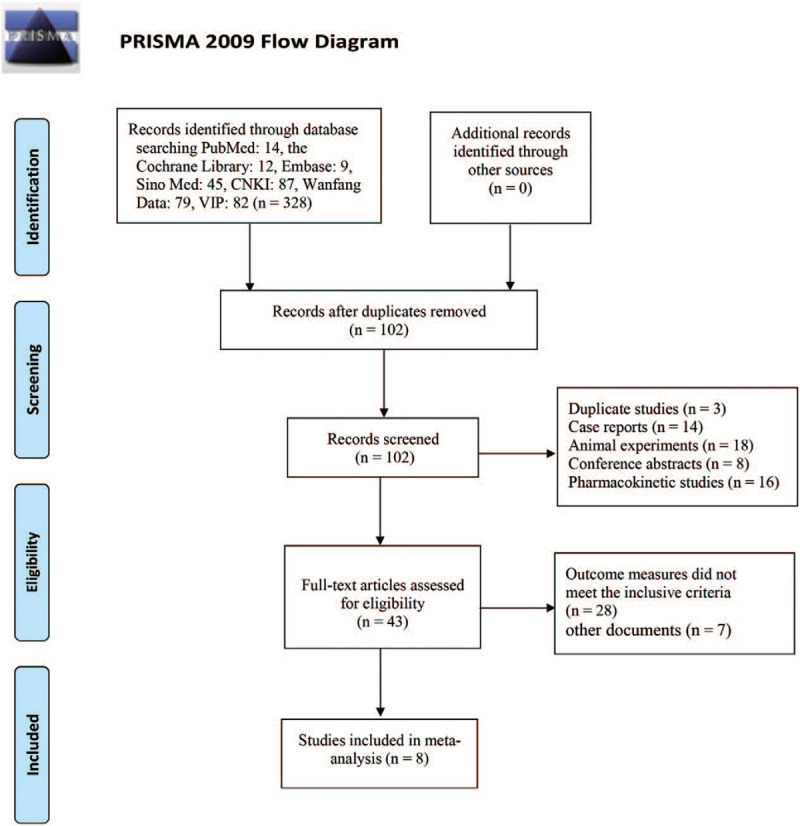
Flow-process diagram of literature retrieval.

A total of 328 related literatures meeting our inclusion criteria were obtained in the initial examination. Two hundred twenty six of them were excluded because of duplication. We reviewed the titles and abstracts of a total of 102 related literatures. Fifty nine articles were excluded because of the following reasons: case reports (n = 14), animal experiments (n = 23), conference abstracts (n = 8), pharmacokinetic studies (n = 20). We reviewed the full text of the remaining 43 articles. Thirty five of them which did not meet the inclusion criteria were further excluded because of the following reasons: outcome measures did not meet the inclusive criteria (n = 28), other documents (n = 7). After dressing by screening, 8 RCTs^[15–18,21–24]^ containing 556 patients were finally included. The process and results of literature screening are shown in Fig. 1.

### 3.2 Study characteristics

**Figure 2:**
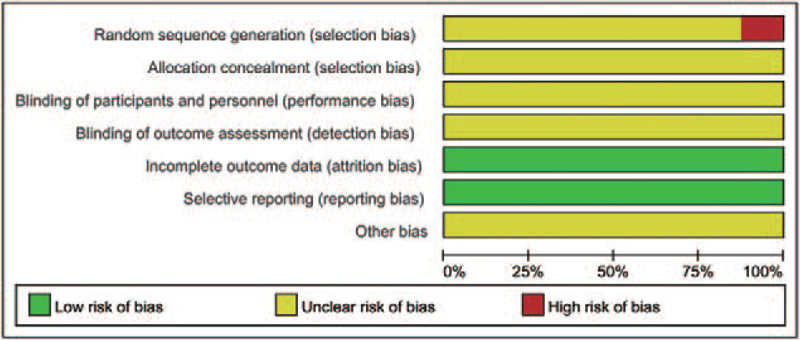
Risk of bias.

**Figure 3:**
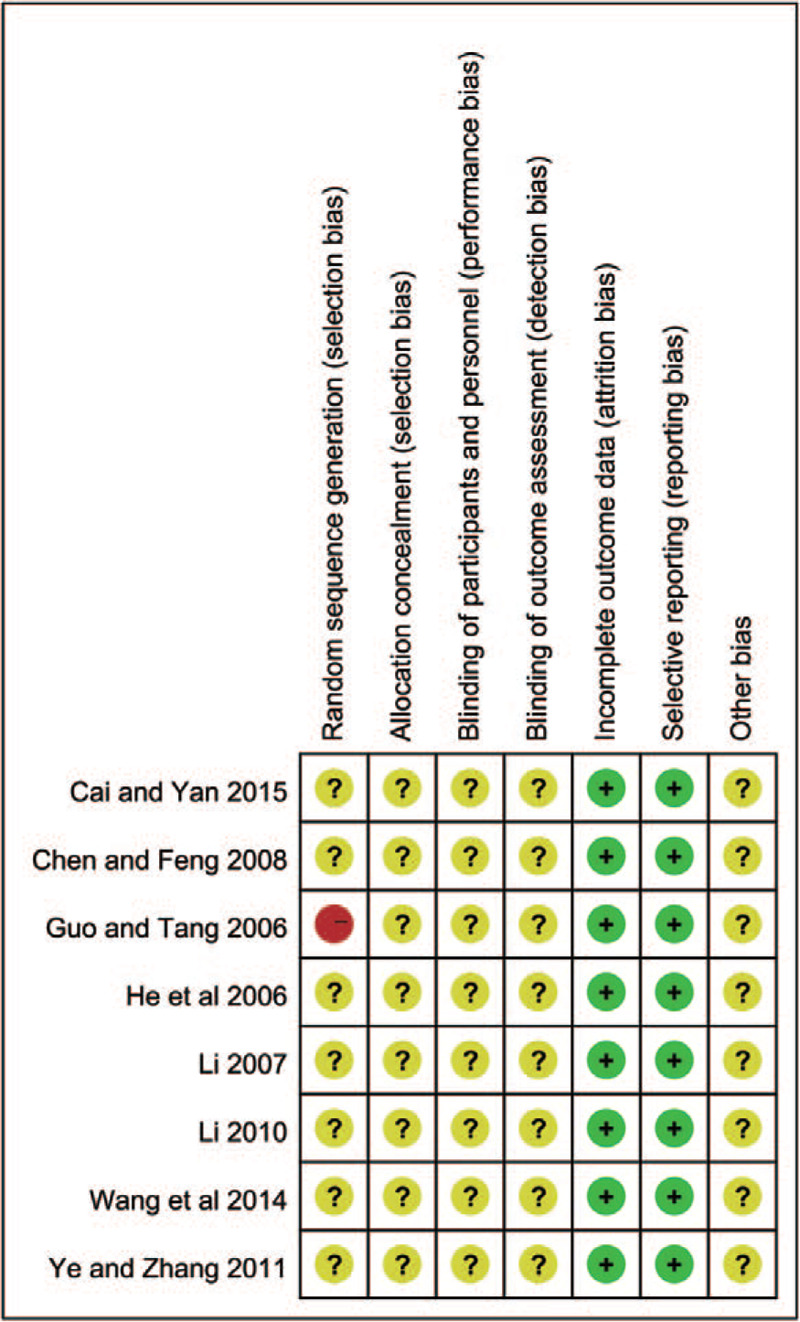
Risk of bias summary and graph.

Eight RCTs^[15–18,21–24]^ containing 556 patients were included in the meta-analysis. Two hundred ninety nine of them belonged to research group while 257 of them belonged to control group. All of them were carried out in the hospitals of China. Included studies were published between 2006 and 2015. The sample sizes ranged from 37 to 117. The basic characteristics of the included studies are shown in Table 1. The studies included just mentioned that they used the random allocation, but they did not mention the specific method of grouping, and none of the included studies discussed about the specific allocation concealment or blinding method. The assessment of bias risk and quality is shown in Figs. 2 and 3.

**Table 1:** Basic features of 8 studies fulfilling inclusion criteria.

Study	Sample size (T/C)	Age (y) range, mean	Intervention	Control	Course of treatment	Outcomes
Cai and Yan, 2015	60 (30/30)	T: 64–79 C: 64–78	GDI (20 mL, ivgtt, qd) + control	Benazepril (10 mg, po, qd)	4 weeks	BUN, 24 h UTP, Scr, Ccr, SBP, DBP, PV, Hct, Hb, WBHSV, WBMSV, WBLSV, RBCAI
Chen and Feng, 2008	68 (35/33)	T: 44–75 C: 43–74	GDI (20 mL, ivgtt, qd) + control	Valsartan (80–160 mg, po, qd)	4 weeks	BUN, 24 h UTP, Scr,
Guo and Tang, 2006	11 (78/39)	T: 59.1 ± 9.8 C: 58.3 ± 10.1	GDI (20 mL, ivgtt, qd) + control	Conventional treatment (no specific details)	4 weeks	Clinical efficacy, PV, Fib, Hct, PAGT, WBV
He et al., 2006	37 (18/19)	T: 45–70 C: 45–70	GDI (20 mL, ivgtt, qd) + control	Benazepril (10 mg, po, qd)	4 weeks	BUN, 24h UTP, Ccr, clinical efficacy
Li, 2007	52 (26/26)	T: 45–70 C: 45–70	GDI (20 mL, ivgtt, qd) + control	Benazepril (10 mg, po, qd)	4 weeks	24h UTP, Scr, clinical efficacy, TG, PV
Li, 2010	90 (45/45)	T: 64.2 ± 10.1 C: 63.8 ± 9.7	GDI (20 mL, ivgtt, qd) + control	Amlodipine Besylate (2.5–10 mg, po, qd)	1 month	BUN, Scr, malb
Wang et al., 2014	78 (40/38)	T: 69.1 ± 3.5 C: 66.1 ± 4.1	GDI (30 mL, ivgtt, qd) + control	Conventional treatment (no specific details)	3 weeks	BUN, 24h UTP, Scr, MAP, Ccr, TC, TG, LDL-C, HDL-C, Hb
Ye and Zhang, 2011	54 (27/27)	T: 60–82 C:60–82	GDI (20 mL, ivgtt, qd) + control	Benazepril (10 mg, po, qd)	4 weeks	24h UTP, BUN, Scr, SBP, DBP, WBHSV, WBMSV, WBLSV, PV, Hct, RBCAI

24 h UTP = 24 hours urinary total protein, BUN = blood urea nitrogen, C = control group, Ccr = creatinine clearance rate, DBP = diastolic blood pressure, Fib = fibrinogen, GDI = Ginkgo leaf extract and dipyridamole injection, Hb = hemoglobin, Hct = hematocrit, LDH-C = lactate dehydrogenase C, LDL = low-density lipoprotein, malb = urinary microalbumin, MAP = mean arterial pressure, PAGT = platelet aggregation rate, PV = plasma viscosity, RBCAI = red cellaggregation index, SBP = systolic blood pressure, Scr = serum creatinine, T = treatment group, TC = total cholesterol, TG = total triglycerides, WBHSV = high shear viscosity of whole blood, WBLSV = low shear viscosity of whole blood, WBMSV = middle shear viscosity of whole blood, WBV = whole blood viscosity.

### 3.3 Primary outcome measures

#### 3.3.1 24-hour urinary total protein (24 h UTP, g/d)

**Figure 4:**
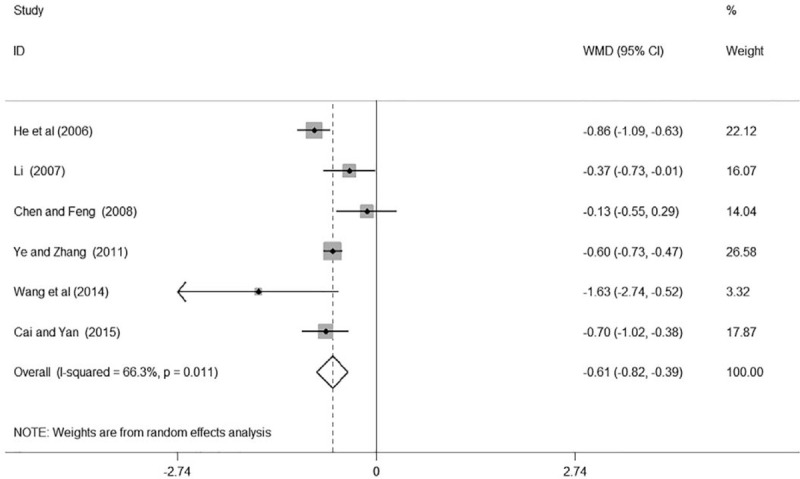
Forest plot of 24 h UTP of Ginkgo leaf extract and dipyridamole injection combined with regular treatment versus regular treatment. 24 h UTP = 24 hours urinary total protein.

Six studies^[15–18,23,24]^ reported the result of the 24 h UTP in patients with hypertensive renal impairment. Five of them^[15–16,18,23,24]^ found GDI plus conventional treatment had a significant decrease of 24 h UTP in patients with hypertensive nephropathy while the remaining one of them^[17]^ reported non-significant association. In total, the meta-analysis indicated that GDI combined with conventional medicine had a significant reduction of 24 h UTP compared with using conventional medicine alone in patients with hypertensive nephropathy (WMD –0.61, 95% CI: –0.82, –0.39; *k* = 6, *P* ≤ .001, Fig. 4).

#### 3.3.2 Blood urea nitrogen (BUN, mmol/L)

**Figure 5:**
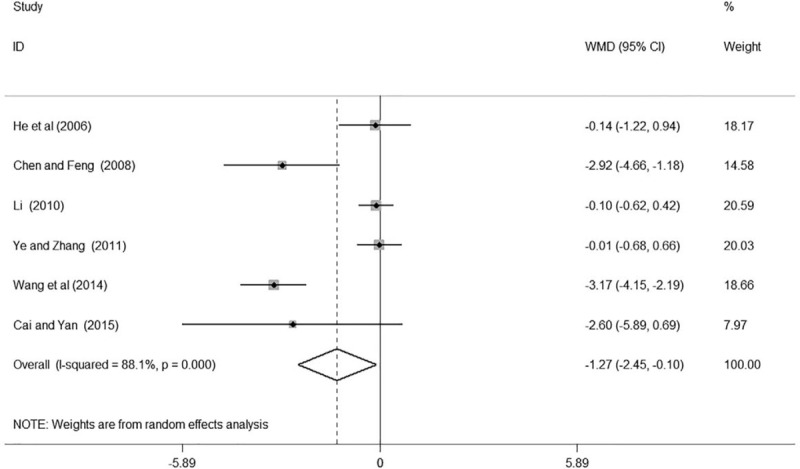
Forest plot of BUN of Ginkgo leaf extract and dipyridamole injection combined with regular treatment versus regular treatment. BUN = blood urea nitrogen.

Six studies^[15,17–18,22–24]^ reported the result of BUN in patients with hypertensive renal impairment. Two of them^[17,23]^ found GDI plus conventional treatment had a significant decrease of BUN in patients with hypertensive nephropathy while the remaining 4 of them^[15,18,22,24]^ reported non-significant association. In total, our meta-analysis indicated that GDI combined with conventional medicine had a significant reduction of BUN compared with using conventional medicine alone in patients with hypertensive nephropathy (WMD 1.27, 95% CI: –2.45, –0.10; *k* = 6, *P* = .033, Fig. 5).

#### 3.3.3 Serum creatinine (Scr, μmol/L)

**Figure 6:**
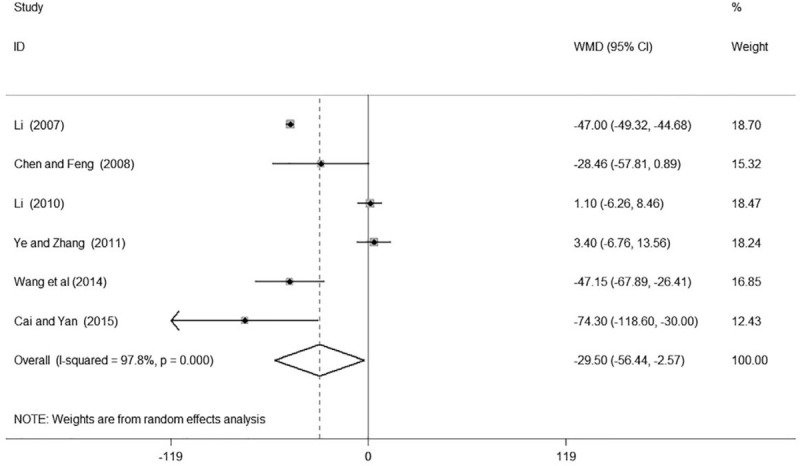
Forest plot of Scr of Ginkgo leaf extract and dipyridamole injection combined with regular treatment versus regular treatment. Scr = serum creatinine.

Six of the included studies^[16–18,22–24]^ involving 402 participants reported the results of Scr in patients with hypertensive nephropathy. Three of them^[16,23,24]^ found GDI plus conventional treatment had a significant decrease of Scr in patients with hypertensive nephropathy while the remaining 3 of them^[17–18,22]^ reported non-significant association. In total, the meta-analysis indicated that GDI combined with conventional medicine had a significant reduction of Scr compared with using conventional medicine alone in patients with hypertensive nephropathy (WMD –29.50, 95% CI: –56.44, –2.56; number of estimates [*k*] = 6, *P* = .032, Fig. 6).

#### 3.3.4 Secondary outcome measures

Three of the included studies found^[15,23,24]^ GDI plus conventional treatment had a significant increase of Ccr in patients with hypertensive nephropathy (WMD 11.11, 95% CI: 3.17, 19.06; *k* = 3, *P* = .006, Table 2). Three of the included studies^[18,23,24]^ found GDI plus conventional treatment had a significant decrease of MAP in patients with hypertensive nephropathy (WMD –8.837, 95% CI: –11.44, –6.24; *k* = 3, *P* ≤ .001, Table 2). Three of the included studies^[15–16,21]^ found GDI plus conventional treatment had a significant increase of clinical efficacy in patients with hypertensive nephropathy (RR 1.34, 95% CI: 1.156, 1.545; *k* = 3, *P* ≤ .001, Table 2).

**Table 2:** Meta-analyses of secondary outcome measures.

Outcome measure	Reports (n)	WMD/RR	95% CI	*I*^2^	*P*
Ccr	3	11.11	(3.17, 19.06)	80.1%	.006
MAP	3	–8.84	(–11.44, –6.24)	3.8%	<.001
Clinical efficacy	3	1.34	(1.16, 1.55)	0.0%	<.001

Ccr = creatinine clearance rate, MAP = mean arterial pressure, RR = relative risk, WMD = weighted mean difference.

#### 3.3.5 Safety

Six of the included studies^[15,17–18,21–23]^ reported adverse events. There were 3 cases of slight epigastric discomfort and nausea, 1 case of slight headache, and 1 case of red spot papule with pruritus. All symptoms recovered after symptomatic treatment without affecting the treatment.^[15]^ Five studies^[17–18,21–23]^ reported that there were no adverse events related to GDI combined with antihypertensive therapy. The remaining 2 studies^[16,24]^ did not mention the adverse events.

#### 3.3.6 Sensitivity analysis and publication bias

We found high heterogeneity according to the test for heterogeneity (24 h UTP: *I*^*2*^ = 66.3%, BUN: *I*^*2*^ = 88.1%, Scr: *I*^*2*^ = 97.8%, Figs. 4–6), and the random effect model was used in this meta-analysis. We used the sensitivity analysis to judge the stability of the results, and no obvious effect on the pooled association estimate was found (Figures S1, S2, and S3, Supplemental Digital Content). Funnel plots and Egger tests were used to determine the publication bias by us, and no publication bias was found (Egger test: 24 h UTP: *P* = .923, BUN: *P* = .208, Scr: *P* = .342, Figures S4, S5, S6, Supplemental Digital Content and Table S3, Supplemental Digital Content).

## 4 Discussion

This study systematically evaluated the efficacy and safety of GDI combined with conventional antihypertensive drugs on renal function and related outcome indicators in patients with hypertensive renal impairment. Meanwhile, the results showed that the level of 24 h UTP, BUN, Scr, and MAP in GDI combined with regular treatment group were significantly lower than that in the control group. The level of Ccr and clinical efficacy in combination group were significantly higher than control group, and no serious adverse events were found in this meta-analysis. It suggested that GDI combined with antihypertensive drugs had a protective effect on the renal function of patients with hypertensive renal injury and can be widely used in clinical treatment.

GDI is a compound preparation containing total flavonoids and dipyridamole. Flavonoids of *G biloba* L. are extracted from *G biloba* L. which have the function of dilating vessels.^[25,26]^ Dipyridamole can inhibit the aggregation of platelet. 24 h UTP, BUN, Scr, and Ccr are key indicators which reflect renal function in clinical trials.

Proteinuria is an important biomarker used to assess the severity of renal injury.^[27]^ Even after a good control of blood pressure, reduction of proteinuria still has a very significant predictive value to slow down the progression of hypertensive nephropathy.^[28]^ We should pay special attention to the degree of proteinuria for assessment of the renal function. In our meta-analysis, we found the combination treatment of GDI with antihypertensive drugs can reduce the 24 h UTP effectively. GDI can also reduce the damage of glomerulus by reducing oxygen free radicals, improve the permeability of glomerular basement membrane and reduce the loss of plasma protein.^[29,30]^ The combination of GDI and antihypertensive drugs may produce a synergistic effect of lowering the 24 h UTP through these mechanisms.

BUN is another important biomarker detecting renal function. It is a nitrogen-containing compound other than protein in the plasma, which is filtered from the glomerulus and discharged from the body. It is used as an index to judge the glomerular filtration function. Our meta-analysis found that the combination treatment of GDI with antihypertensive drugs can reduce BUN effectively. Renal damage is a major complication of hypertension. It is mainly due to the increase of effective renal filtration pressure caused by hypertension and glomerular hyperperfusion and hyperfiltration caused by the increase of glomerular plasma flow.^[31,32]^ GDI can prevent angiotensinogen from converting into angiotensin I and II by inhibiting angiotensin-converting enzyme, thus reducing the damage of renal interstitium caused by high pressure and hyperperfusion.

The level of Scr can reflect renal function accurately and sensitively and it has a good clinical value in the evaluation of renal function in hypertensive nephropathy.^[33]^ Our analysis indicated that GDI combined with antihypertensive drugs can significantly decrease Scr. GDI has the functions of expanding renal blood vessels, enhancing renal blood flow, improving renal microcirculation, blocking renal fibrosis, reducing Scr, BUN, etc.^[34]^ The combination of GDI with conventional therapy may have a synergistic effect of reducing Scr through above mechanisms. In addition, we found the combination treatment can improve the Ccr, MAP, and clinical efficacy of patients with hypertensive renal damage. The results of this study suggested that the antihypertensive effect of GDI may be achieved by improving renal microcirculation and reducing blood pressure. At present, angiotensin receptor blocker and ACE inhibitor are the most commonly used drugs for hypertensive renal injury, but some patients did not response well and even had serious side effects.^[35]^ Our study found the combination treatment was better than using antihypertensive drugs alone for renal protection. It can enrich the treatment measures and prolong the life span of patients with hypertensive nephropathy.

There were some limitations in this meta-analysis as follows: there were only 8 eligible RCTs included, and we were not able to conduct subgroup analysis according to the drug dosage, follow-up time, and the type of combination drugs. Most of the included studies did not specifically report the random method, allocation concealment and blinding method, the quality score of the included articles was relatively low. The types of antihypertensive drugs and the duration of intervention of the included studies were not uniform and the population was all from China, which may influence the generalization of our conclusion. The antihypertensive drugs used in conventional treatment of the included studies only covered Benazepril, Amlodipine Besylate, Valsartan, and the effects of GDI combined with other types of antihypertensive drugs for treating renal damage are unclear.

Our meta-analysis showed that GDI combined with regular treatment can improve 24 h UTP, BUN, Scr, Ccr, MAP, and clinical efficacy in patients with hypertensive nephropathy. It suggested that the combination treatment of GDI with antihypertensive drugs was effective and safe on hypertensive kidney damage and can become a supplementary treatment for hypertensive nephropathy (Table S4, Supplemental Digital Content).

## Author contributions

**Conceptualization:** Dinala·Jialiken, Chong Zou.

**Data curation:** Shuai Ren, Junyao Xu, Li-chao Qian.

**Formal analysis:** Shuai Ren, Lihua Wu, Junyao Xu.

**Investigation:** Li-hua Wu.

**Methodology:** Li-hua Wu.

**Project administration:** Lichao Qian, Chong Zou, Li-hua Wu.

**Resources:** Li-hua Wu.

**Software:** Li-hua Wu.

**Supervision:** Dinala·Jialiken, Chong Zou, Li-hua Wu.

**Validation:** Li-hua Wu.

**Visualization:** Jun-yao Xu.

**Writing–original draft:** Lichao Qian, Jun-yao Xu.

**Writing–review & editing:** Dinala·Jialiken, Shuai Ren, Chong Zou.

## Supplementary Material

Supplemental Digital Content

## Supplementary Material

Supplemental Digital Content

## Supplementary Material

Supplemental Digital Content

## Supplementary Material

Supplemental Digital Content

## Supplementary Material

Supplemental Digital Content

## Supplementary Material

Supplemental Digital Content

## Supplementary Material

Supplemental Digital Content

## Supplementary Material

Supplemental Digital Content

## Supplementary Material

Supplemental Digital Content

## Supplementary Material

Supplemental Digital Content
