# Supplementary material for: Combined therapy of hypertensive nephropathy with ginkgo leaf extract and dipyridamole injection and antihypertensive drugs: A systematic review and meta-analysis
Source: Medicine (Baltimore). 2021 May 14;100(19):e25852. doi: 10.1097/MD.0000000000025852 (PMC8133258; doi:10.1097/MD.0000000000025852)
Supplement: Supplemental Digital Content [file medi-100-e25852-s001.docx]

# Table S1. Search strategies used for PubMed and other English language databases.

| #1 | Hypertension, Renal [MeSH] OR Hypertension, Renovascular [MeSH] OR Hypertensive Nephropathy [Title/Abstract] OR Hypertensive Kidney Lesion [Title/Abstract] OR Hypertensive Renal Damage [Title/Abstract] OR Hypertension-induced renal damage [Title/Abstract] OR Hypertension [Mesh] OR Essential Hypertension [Title/Abstract] OR Primary Hypertension [Title/Abstract] OR EH [Title/Abstract] OR Blood Pressure [Title/Abstract] |
| --- | --- |
| #2 | Ginkgo leaf extract and dipyridamole [Mesh] OR Ginkgo leaf extract and dipyridamole [Title/Abstract] OR Ginkgo Extract [Title/Abstract] OR Ginkgo biloba Leaf [Title/Abstract] OR Ginkgo biloba [Title/Abstract] OR Yinxingdamo [Title/Abstract] OR Ginkgo-damole [Title/Abstract] OR Xingding [Title/Abstract] OR GLEDI [Title/Abstract] OR GDI [Title/Abstract] |
| #3 | Randomized controlled trial [Title/Abstract] OR Controlled clinical trial [Title/Abstract] OR Randomized [Title/Abstract] |
| #4 | #1 AND #2 AND #3 |
