# Supplementary material for: Combined therapy of hypertensive nephropathy with ginkgo leaf extract and dipyridamole injection and antihypertensive drugs: A systematic review and meta-analysis
Source: Medicine (Baltimore). 2021 May 14;100(19):e25852. doi: 10.1097/MD.0000000000025852 (PMC8133258; doi:10.1097/MD.0000000000025852)
Supplement: Supplemental Digital Content [file medi-100-e25852-s003.docx]

# Table S2. Search strategies used for CNKI and other Chinese language databases.

| #1 | Gao Xue Ya Shen Yan [MeSH Terms] OR Gao Xue Ya Shen Bing [Title/ Abstract] OR Gao Xue Ya Shen Sun Hai OR Gao Xue Ya |
| --- | --- |
| #2 | Yinxingdamo [MeSH Terms] OR Yinxingdamo Preparations [Title/Abstract] OR Yinxingdamo Zhusheye [Title/Abstract] OR Zhusheyong Yinxingdamo [Title/Abstract] OR Xingding Zhusheye [Title/Abstract] OR Zhusheye Xingding [Title/Abstract] |
| #3 | Suiji Duizhao Shiyan [Title/Abstract] OR Duizhao Linchuang Shiyan [Title/Abstract] OR Suiji [Title/Abstract] |
| #4 | #1 AND #2 AND #3 |
