# Supplementary figures and images for: Combined therapy of hypertensive nephropathy with ginkgo leaf extract and dipyridamole injection and antihypertensive drugs: A systematic review and meta-analysis
Source: Medicine (Baltimore). 2021 May 14;100(19):e25852. doi: 10.1097/MD.0000000000025852 (PMC8133258; doi:10.1097/MD.0000000000025852)

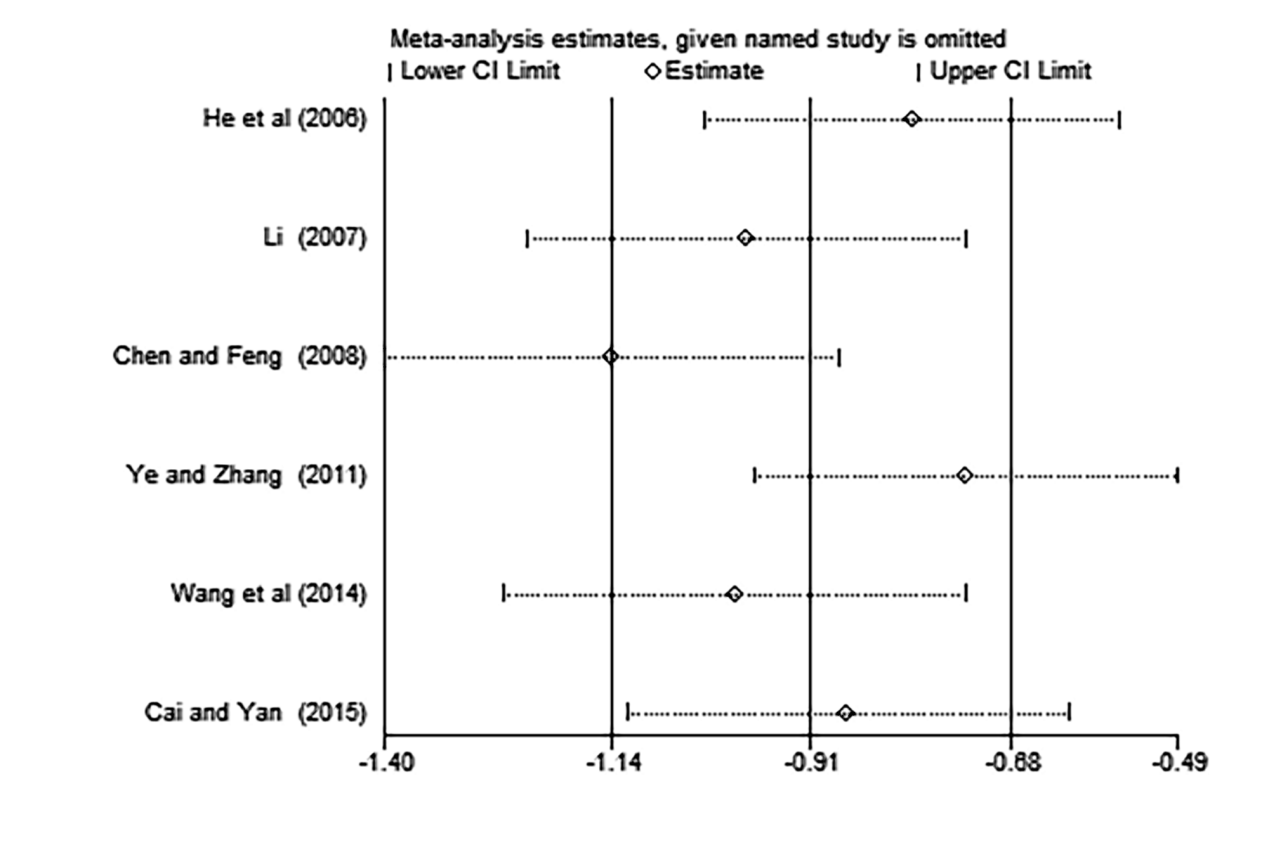


# Figure S1. Sensitivity analysis for 24 h UTP.

Supplement: Supplemental Digital Content [file medi-100-e25852-s004.docx]

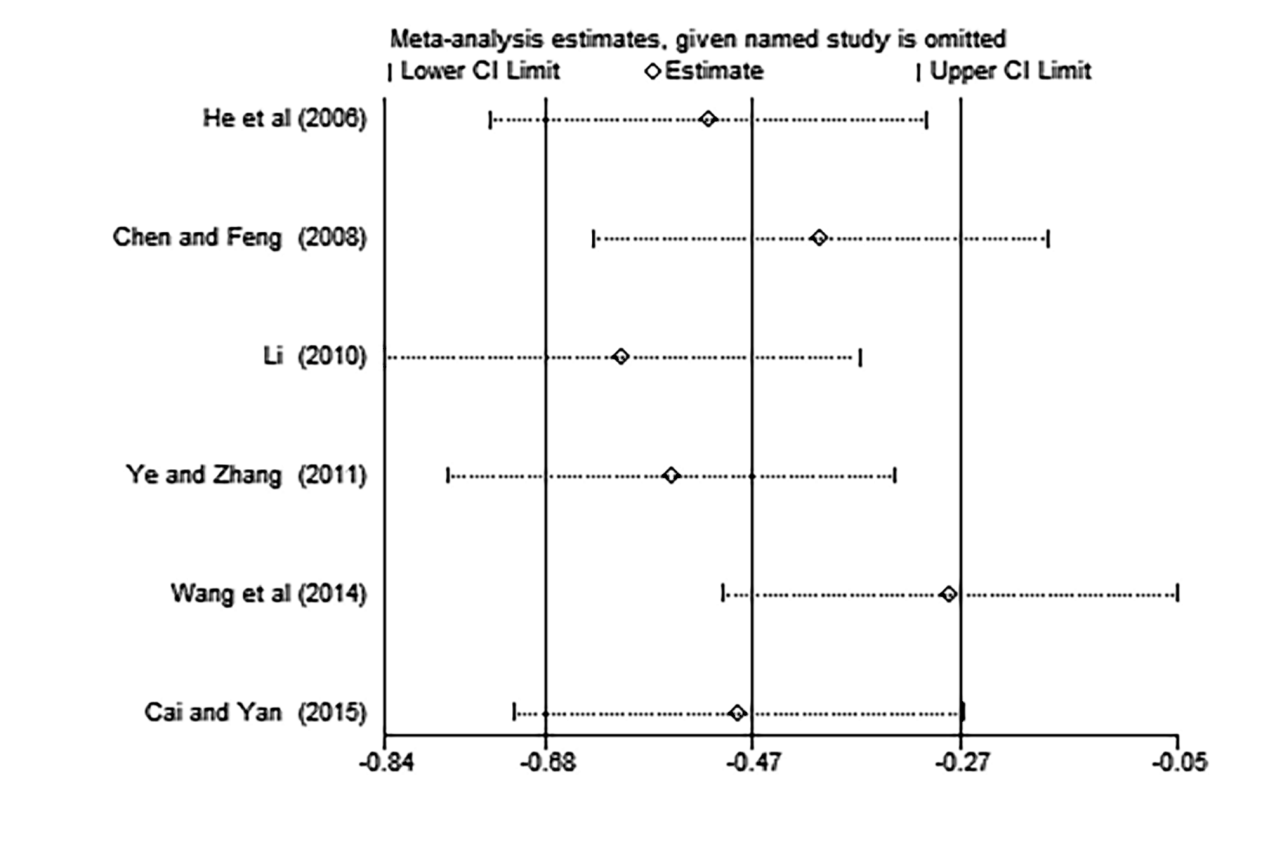


# Figure S2. Sensitivity analysis for BUN.

Supplement: Supplemental Digital Content [file medi-100-e25852-s005.docx]

**
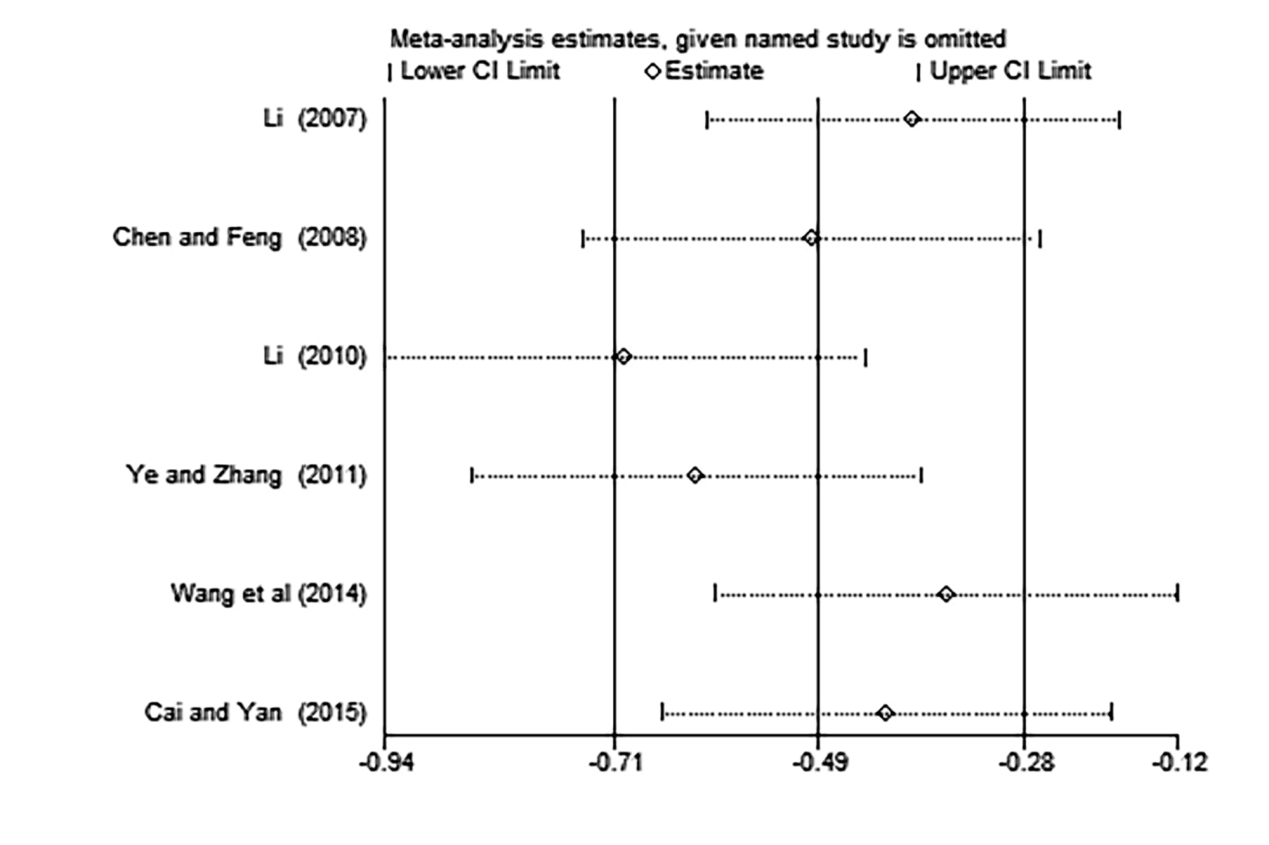
**

# Figure S3. Sensitivity analysis for Scr.

Supplement: Supplemental Digital Content [file medi-100-e25852-s006.docx]

**
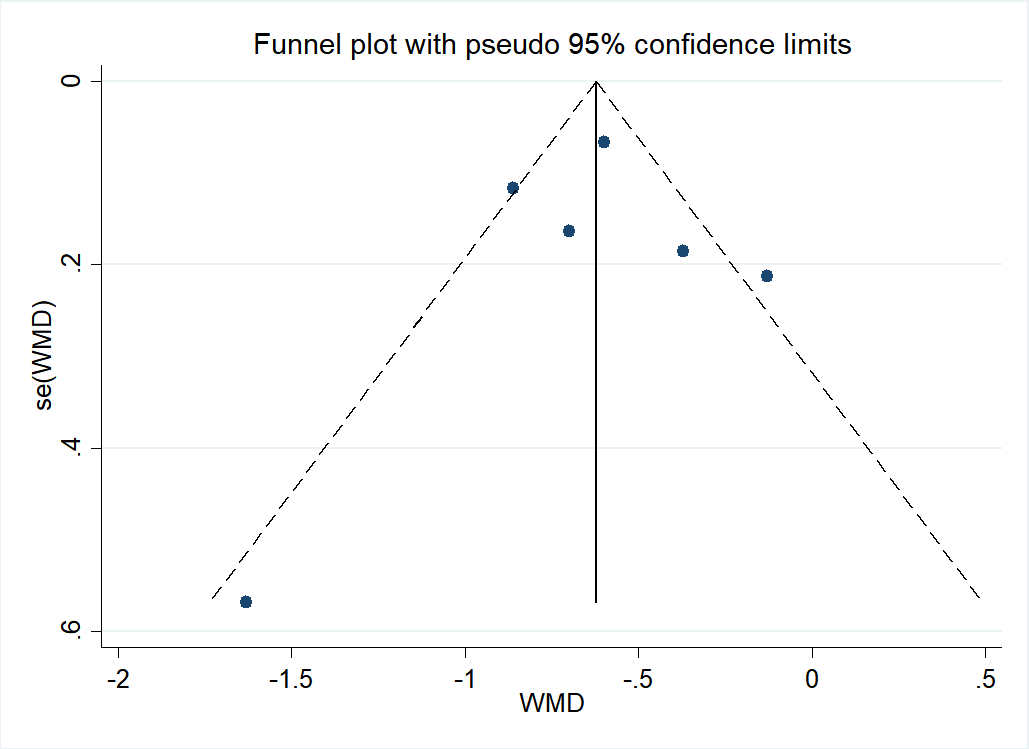
**

# Figure S4. Funnel plot for 24 h UTP.

Supplement: Supplemental Digital Content [file medi-100-e25852-s007.docx]

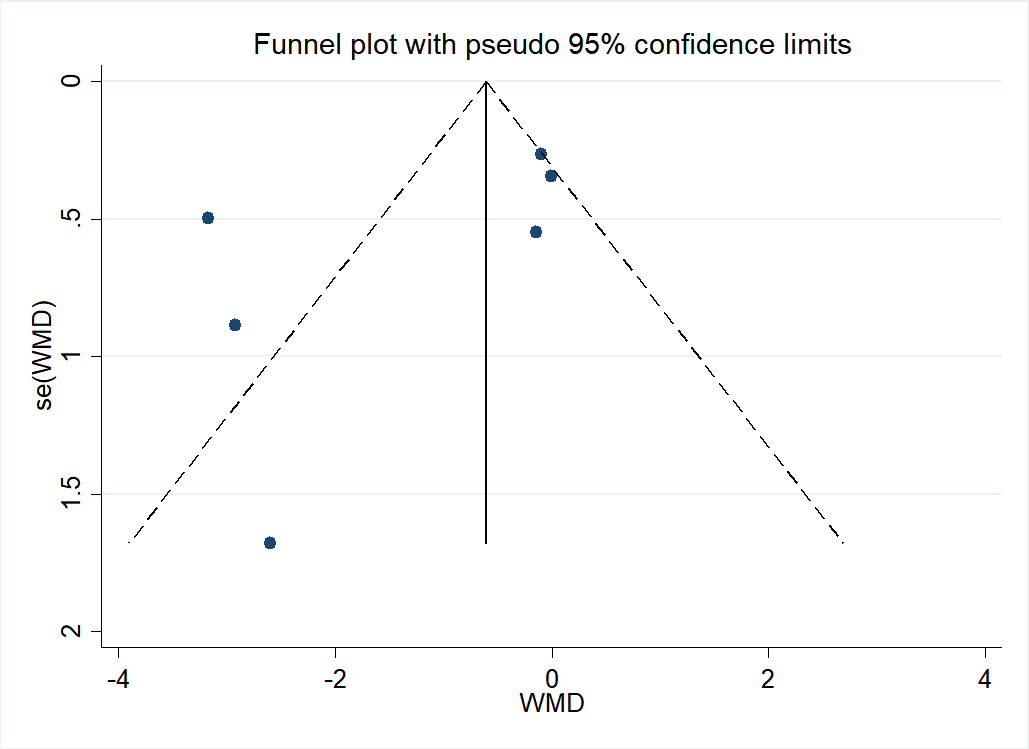


# Figure S5. Funnel plot for BUN.

Supplement: Supplemental Digital Content [file medi-100-e25852-s008.docx]

**
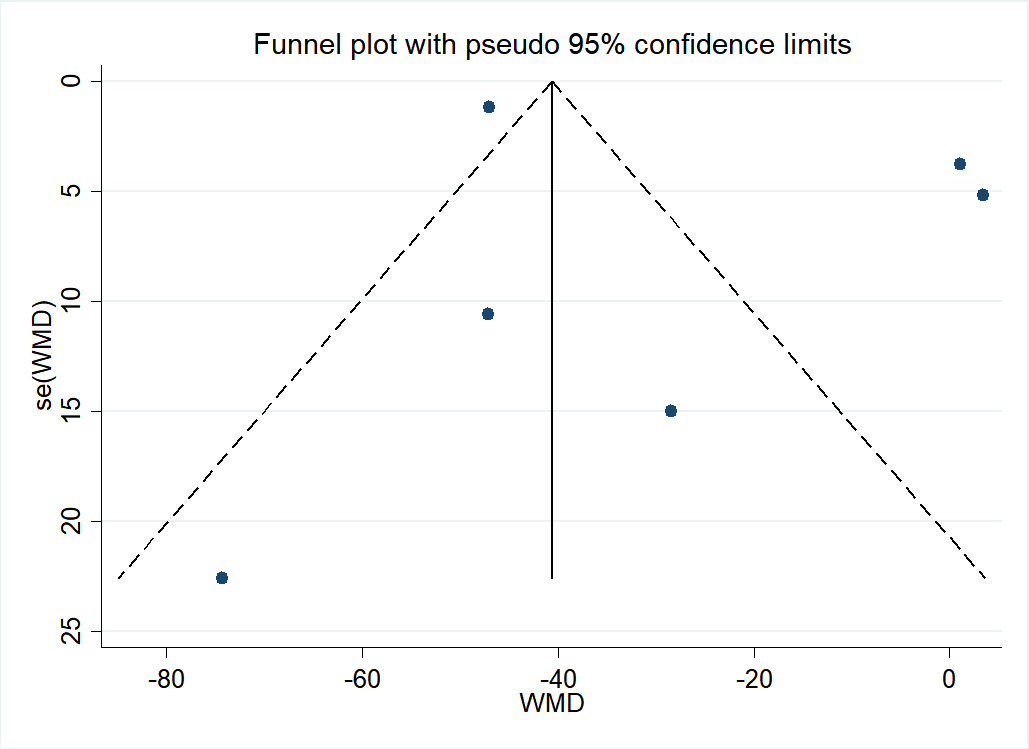
**

# Figure S6. Funnel plot for Scr.

Supplement: Supplemental Digital Content [file medi-100-e25852-s009.docx]
