# Supplementary material for: Combined therapy of hypertensive nephropathy with ginkgo leaf extract and dipyridamole injection and antihypertensive drugs: A systematic review and meta-analysis
Source: Medicine (Baltimore). 2021 May 14;100(19):e25852. doi: 10.1097/MD.0000000000025852 (PMC8133258; doi:10.1097/MD.0000000000025852)
Supplement: Supplemental Digital Content [file medi-100-e25852-s010.docx]

# Table S3. Egger’s test to evaluate publication bias.

| **Outcome** | **Egger’s test (*P*)** |
| --- | --- |
| 24h UTP | 0.923 |
| BUN | 0.208 |
| Scr | 0.342 |
